# Integrated PET-IVIM-DKI MRI for predicting lymphovascular invasion in NSCLC

**DOI:** 10.1186/s13244-025-02078-3

**Published:** 2025-10-30

**Authors:** Qianqian Chen, Nan Meng, Dujuan Li, Xue Liu, Yaping Wu, Yang Yang, Zhun Huang, Zhe Wang, Meiyun Wang, Fangfang Fu

**Affiliations:** 1 Department of Radiology, Zhengzhou University People’s Hospital & Henan Provincial People’s Hospital, Zhengzhou, Henan 450003 China; 2 Department of Pathology, Zhengzhou University People’s Hospital & Henan Provincial People’s Hospital, Zhengzhou, Henan 450003 China; 3 Beijing United Imaging Healthcare Co., Ltd., Beijing, China; 4 Central Research Institute, United Imaging Healthcare Group, Shanghai, China; 5 Biomedical Research Institute, Henan Academy of Sciences, Zhengzhou, Henan 450003 China

**Keywords:** Non-small cell lung cancer, ^18^F-FDG PET/MRI, Intravoxel incoherent motion, Diffusion kurtosis imaging, Lymphovascular invasion

## Abstract

**Objectives:**

To evaluate the potential value of ^18^F-FDG positron emission tomography (PET) and multiparametric MRI (intravoxel incoherent motion, IVIM, and diffusion kurtosis imaging, DKI) in the prediction of lymphovascular invasion (LVI) in non-small cell lung cancer (NSCLC).

**Materials and methods:**

A total of 73 patients with NSCLC who underwent integrated ^18^F-FDG PET/MRI were included. IVIM, DKI, and PET parameters with or without LVI of NSCLC were measured and compared, and the area under the receiver operating characteristic curve (AUC) was used to evaluate the diagnostic efficacy of each parameter. Univariate and multivariate logistic regression models were used to study the optimal combination of PET/MRI parameters for predicting LVI.

**Results:**

PET-derived parameters (SUVmax, MTV, TLG) and IVIM, DKI MRI-derived parameters (ADCstand, D, MK, MD) were significantly different between patients with and without LVI (*p* < 0.05). Multivariate logistic regression analysis showed that MTV and D were independent predictors of LVI, and the combined prediction model of the two parameters had the highest predictive value for the diagnosis of LVI (AUC = 0.841; sensitivity = 63.83%; specificity = 92.31%).

**Conclusion:**

The present study demonstrates that IVIM, DKI, and PET can be utilized to evaluate LVI status in NSCLC, with the combined diagnostic approach of MTV and D showing the highest diagnostic performance, which may provide a novel reference for clinical management.

**Critical relevance statement:**

The performance of metabolic parameters and diffusion parameters in the identification of lymphovascular invasion (LVI) in non-small cell lung cancer (NSCLC) is similar, but the combination of metabolic tumor volume (MTV) and true diffusion coefficient (D) may improve the diagnostic efficacy.

**Key Points:**

- A multimodal PET-MRI model evaluates lymphovascular invasion (LVI) in patients with non-small cell lung cancer (NSCLC).
- Metabolic and diffusion parameters have similar efficacy in predicting LVI in NSCLC.
- The combined metabolic tumor volume and true diffusion coefficient prediction model is the most valuable.

**Graphical Abstract:**

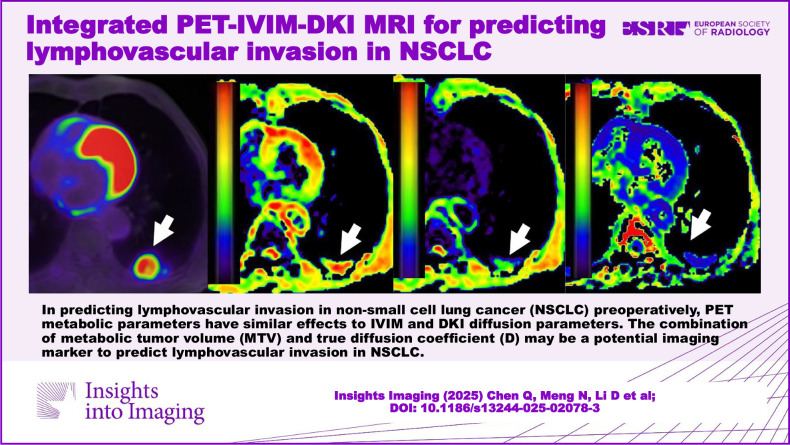

## Introduction

Non-small cell lung cancer (NSCLC) makes up approximately for about 85% [1] of all lung cancer cases, with a 5-year survival rate of only 15%, and is the primary cause of cancer-related deaths globally [2]. Surgery is the preferred course of treatment for NSCLC, and different surgical options can be chosen based on the stage; however, regardless of the type of resection, the treatment effect is not satisfactory. Patients with early-stage NSCLC still have poor surgical survival rates [3]. Therefore, it is critical to identify reliable prognostic factors and intervene in a timely manner. Recent studies have shown that lymphovascular invasion (LVI), as the initial stage of distant and local metastasis, is strongly associated with lymph node [4] metastasis, recurrence, and distant metastasis [5] and prognosis [6] of lung cancer individuals; it has been recommended that LVI be included in TNM [7] staging. Currently, LVI has been included in the latest National Comprehensive Cancer Network guidelines [8] as an indication for adjuvant chemotherapy after surgery. It also holds certain value in guiding postoperative radiotherapy [9] for patients with pN2-III stage NSCLC. According to a retrospective study by Yun et al [10], LVI significantly impacted both overall survival and recurrence-free survival in patients with stage IA non-small cell lung cancer who received sublobar resection and proposed to add lobectomy to patients with confirmed LVI after sublobar resection. Preoperative prediction of LVI plays a pivotal role in guiding therapeutic decision-making for non-small cell lung cancer (NSCLC). For LVI-positive patients, clinicians may consider choosing different operative procedures and administering chemotherapy or targeted therapy. Particularly in early-stage NSCLC patients with LVI, implementing personalized treatment regimens can significantly improve overall survival and reduce recurrence risk.

With the development of related technologies in recent years, medical imaging has become a standard clinical procedure for tumor evaluation, providing more possibilities for personalized medicine than before. However, using traditional assessment methods, it is difficult [11] to accurately and quantitatively evaluate various internal microscopic features, such as microvascular density, cell proliferation, and energy metabolism, which are closely related to tumor prognosis. Previously, several studies [12–15] have predicted the LVI status of NSCLC patients based on CT/PET-CT through morphological features or radiomics models, but it is difficult to show small lesions with ordinary CT, easy to miss the diagnosis (35–45%), and cannot provide hemodynamic and functional status information. At present, radiomics brings challenges to data unification and model generalization due to the differences in image data obtained by different devices and scanning parameters. ^18^F-fluorodeoxyglucose positron emission tomography (^18^F-FDG PET) is a molecular imaging [16] method that focuses on semi-quantitative analysis of changes in glucose metabolism in lesions based on the uptake level of ^18^F-FDG. In NSCLC, certain researchers have seen a positive connection between the SUVmax and pathological risk factors [17], such as LVI [18], and ^18^F-FDG PET/CT can be a valuable predictive biomarker for detecting LVI [19]. However, routine FDG-PET/CT images fail [20] to detect small vascular invasions due to limited spatial resolution and show high false-negative rates (25–30%) for hypometabolic tumors.

   Diffusion-weighted imaging (DWI) is an MRI imaging sequence that reflects the diffuse movement of water molecules [21] in tissues. Previous studies have shown that ADC value, as a diffusion parameter of DWI, is negatively correlated with lymphovascular invasion of breast [22] or cervical [23] cancer to a certain extent. Another study [24] showed that ADCs were associated with tumor aggressiveness. As a quantitative MRI technique based on the Brownian motion of water molecules, intravoxel incoherent motion (IVIM) is an extension of traditional DWI that can reveal information about the microperfusion and diffusion of water molecules in a lesion in a single scan [25], measured by parameters including: (1) true diffusion coefficient (D), which reflects the fluidity of water molecules in tissues and depends on cell structure, curvature of extracellular space, integrity of cell membranes, and viscosity of fluid; (2) fractional perfusion (f), which reflects the relative contribution of microvascular blood flow to DWI signaling; and (3) pseudo-diffusion coefficient (D*), which depends on blood velocity and the length of capillary segments. Since DKI takes into account the non-Gaussian [26] distribution of water molecules in tissue diffusion movement, it can be used to analyze the microstructure of lesions and to perform a noninvasive assessment of tumor heterogeneity and spread movement. Integrated PET/MRI can obtain metabolic, diffusion, and perfusion information at the same time, which might help predict LVI accurately.

   Thus, the primary aim of this study is to determine the predictive value of each quantitative parameter which were obtained from IVIM, DKI and PET for lymphovascular invasion in NSCLC, and to establish an optimal predictive model, thereby providing a reference for individualized clinical treatment of patients with NSCLC.

## Materials and methods

### Patients

**Fig. 1:**
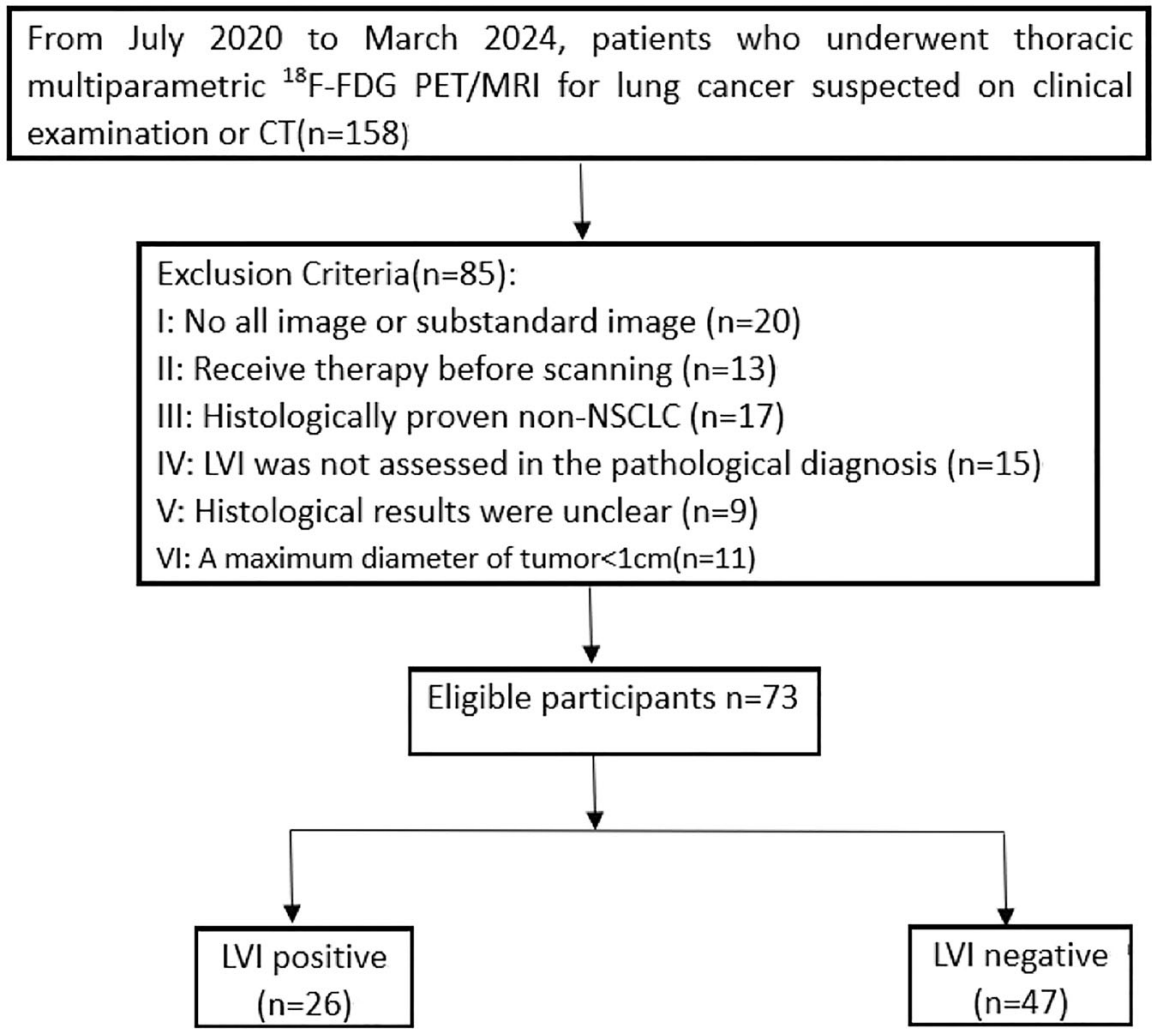
Patient selection flowchart

This study was a prospective cohort study with retrospective analysis, approved by Henan Provincial People’s Hospital Ethics Review Board (NO.2021148). From July 2020 to March 2024, a thoracic multiparametric ^18^F-FDG PET/MRI was performed on 158 patients because a clinical examination or CT scan suggested they might have lung cancer. An informed permission form was signed by each patient before their integrated PET-MRI. Inclusion criteria for this study (Fig 1): (1) All the non-small cell lung cancers were confirmed by pathology after initial PET/MRI scan within 7 days; (2) All patients had not received radiation, chemotherapy, or surgery prior to PET/MRI scan; (3) All patients underwent ^18^F-FDG PET/MRI, and the image data were comprehensive; Exclusion criteria for this study: (1) Poor image quality of PET/MRI images that cannot be used for post-processing analysis; (2) Patients with incomplete relevant clinical and postoperative pathological data; (3) Patients with too small (diameter < 10 mm) lesion or pure ground-glass nodules.

### PET-MRI scanning and image acquisition

The patients in this study underwent lung scans by an integrated 3.0-T PET/MRI (uPMR 790, Union Medical) scanner with a 12-channel phased array body coil. Confide the patient to breathe calmly and use an abdominal band to control movement artifacts and monitor breathing. Fasting for at least 6 h prior to injection to guarantee a fasting blood glucose level below 7.0 mmol/L prior to scanning. The acquisition of images was initiated 60 min after intravenous injection ^18^F-FDG (0.11 mCi/kg), scanning from the lung apex to the diaphragmatic angle. PET scanning and MRI image acquisition took place concurrently during a 27-min timeframe. MRI sequence attenuation correction of γ-rays was performed using a 3D T1-weighted gradient echo sequence with Dixon water-lipid separation approach, and the images were reconstructed using the ordered subset maximum expectation iteration method (OSME). First, T1-weighted imaging (T1WI), T2-weighted imaging (T2WI), and DWI scans were carried out. Next, all slices that included lesions were chosen from the DWI images, and their positions, layer thicknesses, and layer spacing (Table 1) were copied to IVIM and DKI for the corresponding scans.

**Table 1:** Parameters used for MR-IVIM/DKI imaging

Parameters	T1WI	T2WI	DWI	IVIM	DKI
Sequence	2D-FSE	2D-FSE	2D-SS-EPI	2D-SS-EPI	2D-SS-EPI
Orientation	Axial	Axial	Axial	Axial	Axial
TR/TE (ms)	5.06/2.1	3315/87.8	1620/69.6	1620/69.6	1210/86
FOV (cm^2^)	35 × 50	35 × 50	35 × 50	35 × 50	35 × 50
Matrix	303 × 456	264 × 480	202 × 256	202 × 256	100 × 128
Bandwidth (Hz/pixel)	260	260	2370	2370	1630
Slice thickness (mm)	5	5	5	5	5
Interval (mm)	1	1	1	1	1
NEX	2	2	1.8	1, 1, 2, 2, 4, 4, 6, 6, 8, 10	1, 4, 8, 8
b-values (s/mm^2^)	**/**	**/**	0.800	0, 25, 50, 100, 150, 200, 400, 600, 800, 1000	0, 500, 1000, 2000
Respiratory compensation	Yes	Yes	Yes	Yes	Yes
Scan time	14 s	2 min 26 s	2 min 58 s	3 min 38 s	6 min 31 s

*T1WI* T1-weighted imaging, *T2WI* T2-weighted imaging, *DWI* diffusion-weighted imaging, *IVIM* intravoxel incoherent motion, *DKI* diffusion kurtosis imaging, *FOV* field of view, *TR/TE* repetition time/echo time, *NEX* number of excitations

### Image processing and parameter generation

**Fig. 2:**
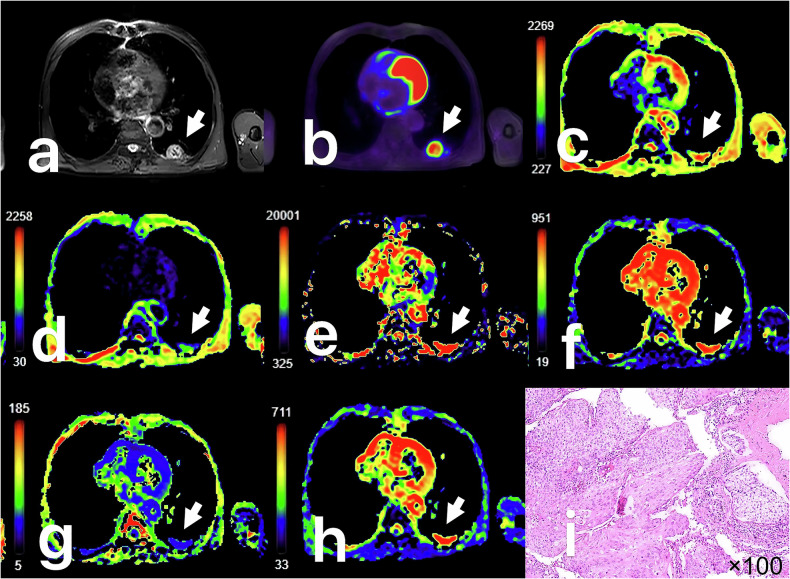
Male, 66 years old, with squamous carcinoma (SCC) in the left upper lobe, size 2.6 × 2.2 × 2.4 cm. **a** Image of axial T2WI with fat saturation pre-pulse. **b** Fusion image of the SUV map and the ultrashort echo time map. **c** Pseudo colored map of ADCstand. **d** Pseudo colored map of D. **e** Pseudo colored map of D*. **f** Pseudo colored map of f. **g** Pseudo colored map of MK. **h** Pseudo colored map of MD. **i** Pathological image that displays positive lymphovascular invasion

**Fig. 3:**
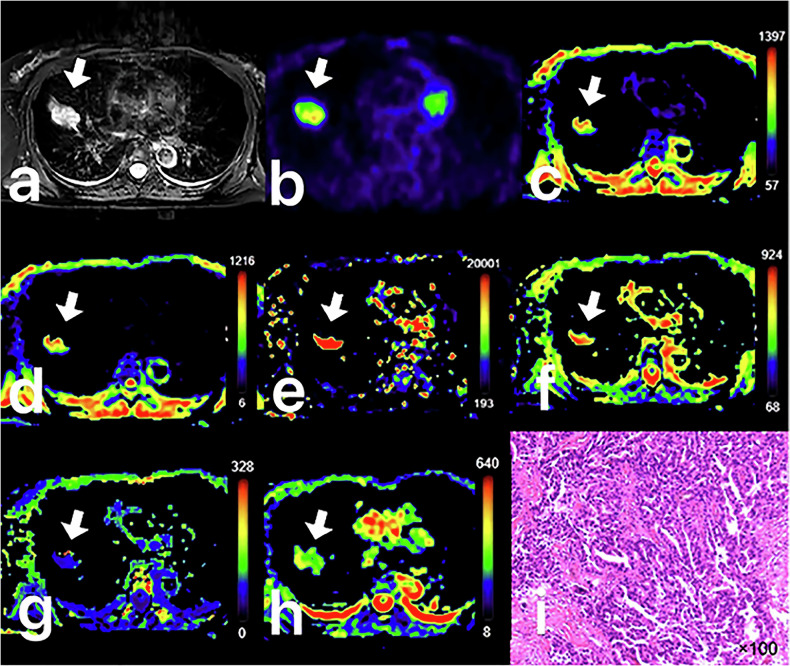
Female, 51 years old, with adenocarcinoma (AC) in the right upper lobe, maximum diameter is 3.1 cm. **a** Image of axial T2WI with fat saturation pre-pulse. **b** Fusion image of the SUV map and the ultrashort echo time map. **c** Pseudo colored map of ADCstand. **d** Pseudo colored map of D. **e** Pseudo colored map of D*. **f** Pseudo colored map of f. **g** Pseudo colored map of MK. **h** Pseudo colored map of MD. **i** Pathological image that displays negative lymphovascular invasion

Each image was imported into the United Imaging Workstation (uWS-MR, UIH) for further manipulation. Without knowing the patient’s information, two radiologists independently (M.N. and F.F.F., with 8 years and 13 years of work experience, respectively) used specialist software in the Advanced Analysis Toolkit to post-process the PET and MRI parameters. Utilizing fusion PET/MRI software, metabolic parameters were post-processed. SUVmax, metabolic tumor volume (MTV), and Total lesion glycolysis (TLG) were computed by [27] automatically extracting the volume of interest (VOI). The IVIM and DKI images were handled with the integrated advanced diffusion analysis program, and the diffusion-weighted imaging (DWI) images were fused with the pseudo-color maps of each parameter. Using axial T1WI, T2WI, and DWI sequences as reference, the physician manually delineated the tumor-containing region of interest (ROI) slice by slice, excluding lymph node tissue and avoiding areas of evident hemorrhage, necrosis, or cystic degeneration. The ROI boundaries were carefully aligned with the corresponding lesion extent on PET images across all slices. At every level, ROIs were gathered, and the mean values of MK, MD, ADCstand, true diffusion coefficient (D), pseudo-diffusion coefficient (D*), and perfusion fraction (f) were noted (Figs. 2, 3). Within 10 days of their PET/MR scan, every patient had a biopsy or surgery to collect specimens.

The formula used to determine the IVIM sequence parameters is presented below. S_b_/S_0_ = (1 − f) × exp(−bD) + f × exp [−b × (D* + D)] for the bi-exponential IVIM model illustrates the relationship between the DWI signal intensity and the b factor. S_b_ represents the signal intensity, and b stands for the sensitivity factor. The diffusion coefficient is represented by D, and the perfusion fraction by f. D* is used to represent the pseudo-diffusion factor [28].

In the DKI model, S_b_ = S_0_ × exp (−b × Dapp + b^2^ × Dapp^2^ × Kapp/6), where Kapp represents the degree of departure from a Gaussian distribution, and Dapp stands for the diffusion coefficient corrected for non-Gaussian deviations. The average Dapp and Kapp values for each direction are shown in MD and MK, respectively [26].

### Pathological diagnosis

Within 10 days of the PET/MRI scan, all specimens were surgically removed and sent to our pathology center. There, they were fixed, dehydrated, dip-waxed, embedded, sectioned, and stained with hematoxylin/eosin (HE) and immunohistochemistry using CD 31 and D2-40. Without knowing the imaging and clinical diagnosis, an experienced pathologist analyzed the samples. The subsequent data were noted: Histopathological type and LVI status. LVI was described as tumor cells in the lymphatic and blood vessel endothelial cell spaces (small arterial/small venous lumen with positive CD 31 brown staining and lymphatic lumen with positive D2-40 brown staining).

### Data analysis

The statistical analysis was carried out using MedCalc, R and SPSS (which was developed by IBM), and *p*-values less than 0.05 were deemed statistically significant. The intraclass correlation coefficient (ICC) was used to assess interobserver agreement (r ≥ 0.75, excellent; 0.60 ≤ r < 0.75, good; 0.40 ≤ r < 0.60, fair; and r < 0.40, poor) [29, 30]. The data were first subjected to the Shapiro–Wilk test to verify that they were normally distributable. Then, Then, those with normal distribution were represented by $\bar{x}\pm s,$ and the independent samples t-test was used for comparison between groups. Those who do not obey the normal distribution are denoted by *M* (*Q1, Q3*) using the Mann–Whitney U test. The area under the curve (AUC) of receiver operating characteristic (ROC) was used to describe the diagnostic performance of each imaging parameter and imaging method, and the DeLong test was used to compare the differences between the two groups. Subsequently, parameters that exhibited statistical significance (*p* < 0.1) in the univariate logistic regression analysis were included in the subsequent multivariate analysis. Multivariate logistic regression models are employed to identify the optimal combination of parameters.

## Results

### Patient characteristics

This study included 73 individuals in total: LVI+ (26) and LVI− (47). Table 2 provides a summary of the patients’ clinical features.

**Table 2:** Patient clinicopathological characteristics

Clinical characteristics		Value
Age (years)		59 ± 9
Sex	Male	34
	Female	39
Smoke	Yes	30
	No	43
Histological type	SCC	7
	AC	66
LVI	+	26
	−	47
Maximum diameter (mm)		27 ± 12
Differentiation grade:		
Well-differentiated		6
Moderately differentiated		41
Poorly differentiated		26

*SCC* squamous cell carcinoma, *AC* adenocarcinoma, *LVI* lymphovascular invasion

### Consistency examine

The measurements made by the two observers resulted in an excellent agreement. The values of the various parameters, which included the MK, MD, ADCstand, D, SUVmax, MTV, and TLG were 0.917, 0.893, 0.862, 0.879, 0.989, 0.978, and 1, respectively.

### Parameter comparison

In the LVI-positive group, SUVmax, MTV, TLG, and MK were significantly higher and ADCstand, D, and MD were significantly lower than in the LVI-negative group (*p* < 0.05 for all) (Table 3).

**Table 3:** Comparison of ^18^F-FDG PET, IVIM and DKI parameters for LVI status

	LVI-positive	LVI-negative	*p*-value
N	26	47	
MK	0.84 ± 0.19	0.70 ± 0.19	0.004
MD (× 10^−3^ mm^2^/s)	2.29 (1.06, 4.77)	2.69 (1.18, 4.58)	0.036
SUVmax (g/cm^3^)	6.07 (0.48, 13.81)	4.19 (0.4, 13.77)	0.014
MTV (cm^3^)	14.75 (2.12, 75.89)	5.59 (0.62, 29.92)	< 0.001
TLG (g)	49.21 (0.69, 259.57)	13.71 (0.68, 193.55)	< 0.001
ADCstand (10^−3^ mm^2^/s)	0.88 (0.25, 1.4)	1.27 (0.25, 4.8)	0.014
D (10^−3^ mm^2^/s)	0.72 (0.01, 1.28)	0.96 (0.06, 3.67)	0.02
D* (10^−3^ mm^2^/s)	75.06 (3.66, 186.8)	68.19 (9.49, 148.01)	0.687
f (%)	41.56 (13.63, 82.06)	41.0 (10.45, 73.38)	0.773

*ADCstand* stand diffusion coefficient, *D* true diffusion coefficient, D* pseudo-diffusion coefficient, *f* perfusion fraction, *MK* mean kurtosis, *MD* mean diffusivity, *LVI* lymphovascular invasion

### Regression analyses

**Fig. 4:**
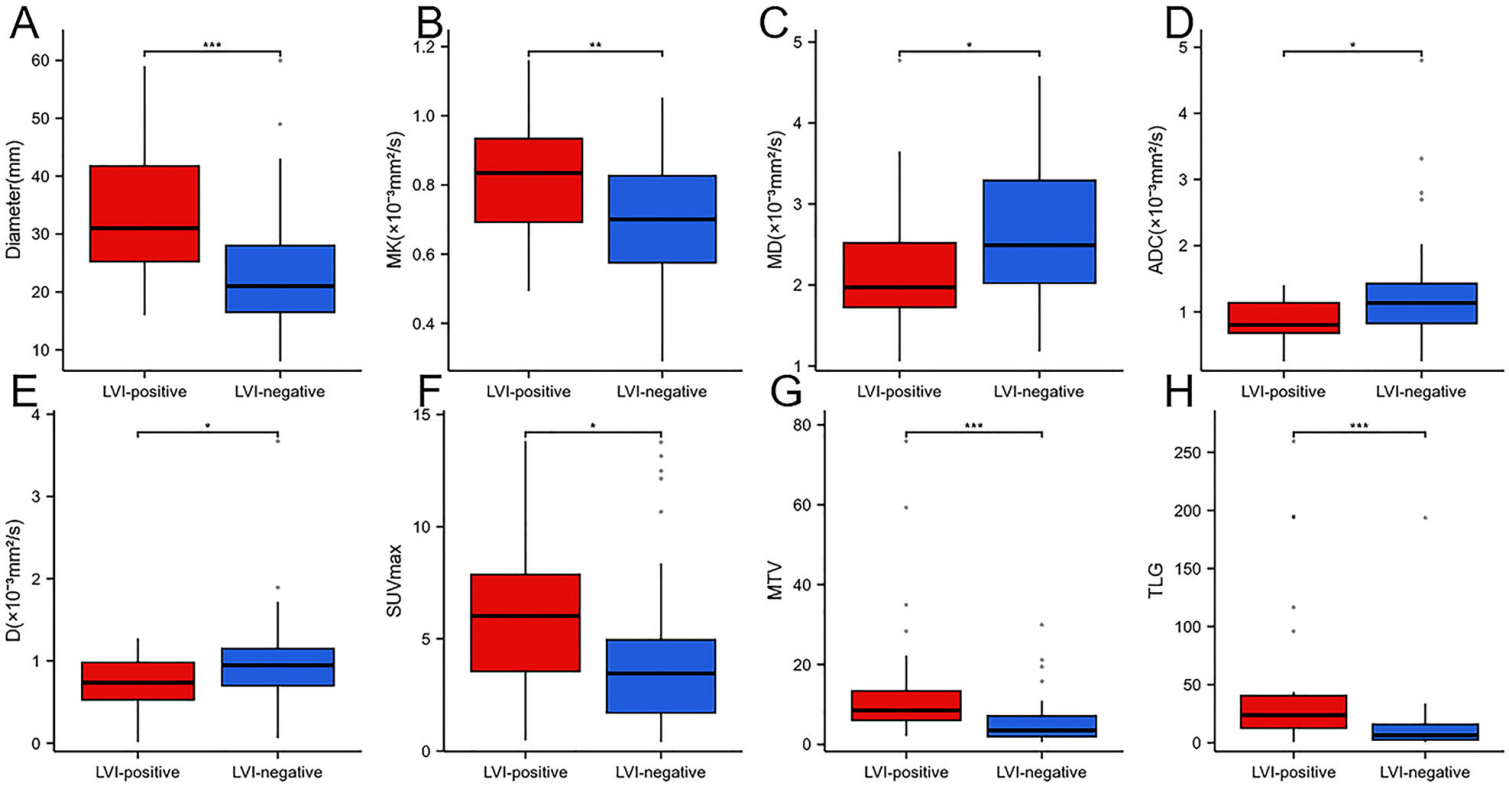
**A**–**H** Showing the comparison of various parameters in the LVI-positive and LVI-negative groups, respectively (diameter, MK, MD, ADCstand, D, SUVmax, MTV and TLG). * stands for *p* < 0.05 and *** for *p* < 0.001

In the identification of LVI (+) and LVI (−), univariate (Table 4) analysis showed that SUVmax, MTV, TLG, ADCstand, D, MK, and MD were predictors, and multivariate analysis revealed that only MTV and D were independent predictors (Fig. 4).

**Table 4:** Univariate and multivariate analyses

Parameters	Univariate analyses		Multivariate analyses	
	OR (95% CI)	*p*-value	OR (95% CI)	*p*-value
SUVmax	1.161 (1.010–1.335)	0.036	1.012 (1.001–1.048)	0.119
MTV	1.11 (1.023–1.2050)	0.012	1.034 (0.997–1.098)	**0.047**
TLG	1.021 (1.001–1.041)	0.035	1.01 (0.977–1.044)	0.566
ADC	0.255 (0.077–0.846)	0.026	0.716 (0.512–0.984)	0.207
D	0.19 (0.046–0.785)	0.022	0.132 (0.027–0.781)	**0.031**
F%	1.002 (0.973–1.031)	0.908	−	−
D*	1.004 (0.993–1.015)	0.512	−	−
MK	0.553 (0.028–19.342)	0.070	0.920 (0.017–49.702)	0.967
MD	0.578 (0.320–1.045)	0.015	0.831 (0.648–0.902)	0.382

All factors with *p* < 0.1 in univariate analyses were included in multivariate regression analyses

The bold typeface in the table indicates the logistic regression analyses with statistical significance (significance level = 0.05)

*SUVmax* maximum standardized uptake value, *MTV* metabolic tumor volume, *TLG* total lesion glycolysis, *ADC* apparent diffusion coefficient, *D* true diffusion coefficient, D* pseudo-diffusion coefficient, *f* perfusion fraction, *MK* mean kurtosis, *MD* mean diffusivity, *OR* odds ratio, *CI* confidence interval

### Diagnostic performance of different parameters and methods

**Fig. 5:**
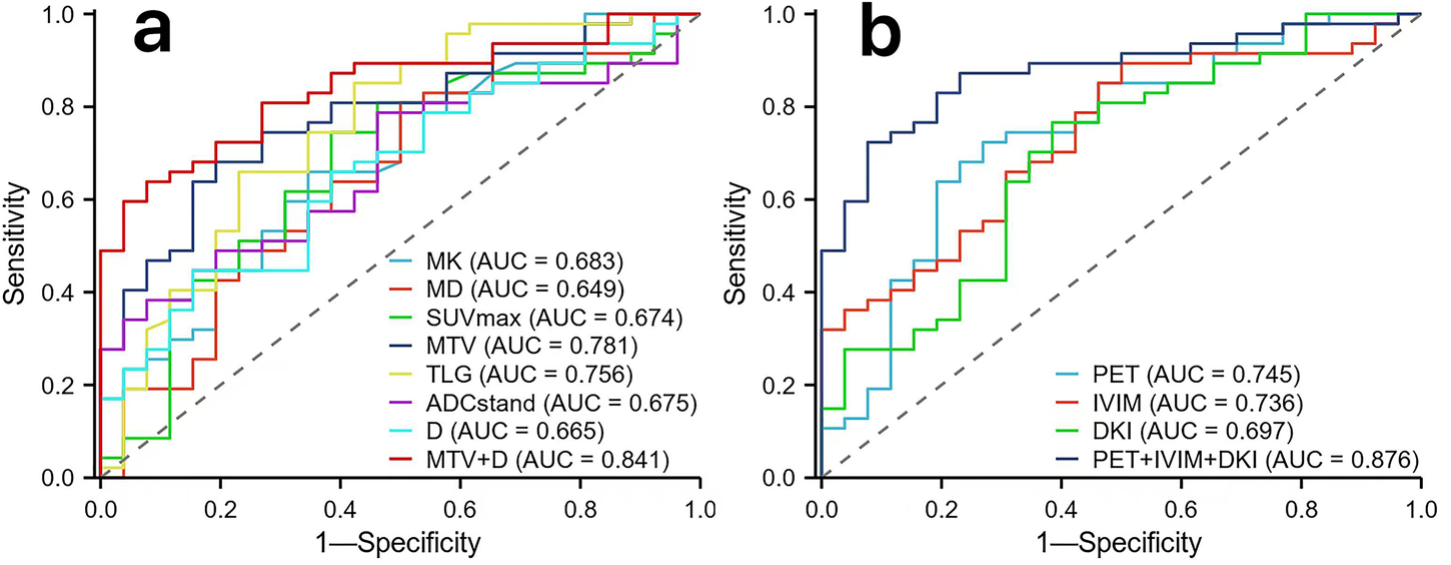
Receiver operating characteristic curves of different parameters and methods, **a** MK, MD, SUVmax, MTV, TLG, ADCstand, D and the combination of independent predictors (MTV + D) (the AUC of each parameter is 0.683, 0.649, 0.674, 0.781, 0.756, 0.675, 0.665, and 0.841, respectively). **b** PET, IVIM, DKI and the combination of three methods (PET + IVIM + DKI) (the AUC of each parameter is 0.745, 0.736, 0.697, and 0.876, respectively)

The combination of independent predictors (MTV + D) performed best in assessing the LVI status ((AUC) of 0.841; sensitivity of 63.83%; specificity of 92.31%); AUC(MTV + D) > AUC(MTV) > AUC(TLG) > AUC(MK) > AUC(ADCstand) > AUC(SUVmax) > AUC(D) > AUC(MD) (AUC = 0.841, 0.781, 0.756, 0.683, 0.675, 0.674, 0.665 and 0.649). The diagnostic efficiency of (MTV + D) is significantly better than MK, ADCstand, SUVmax, D, and MD. While there was no significant difference in AUC, it was more specific than MTV and TLG. The diagnostic capabilities of different methods to predict LVI positivity and LVI positivity are as follows: AUC (IVIM + DKI + PET) > AUC (PET) > AUC (IVIM) > AUC (DKI) (AUC = 0.876, 0.745, 0.736, and 0.697). (IVIM + DKI + PET) AUC (Fig. 5) was significantly greater than that of any single test method, and there was no statistical difference between the three test methods (Table 5).

**Table 5:** ROC analysis of the diagnostic performance for different parameters and methods alone or in combination for predicting LVI status

	AUC (95% CI)	Cutoff	Specificity (%)	Sensitivity (%)	FPR	FNR	Comparison with combined diagnosis
LVI (+) vs LVI (−)							
Parameters							
MK	0.683 (0.555–0.811)	0.78	65.39	65.96	34.61	34.04	Z = 2.3301 *p* = 0.0198
MD	0.649 (0.513–0.785)	1.9	50	80.85	50	19.15	Z = 2.4565 *p* = 0.014
ADC	0.675 (0.552–0.799)	0.81	53.85	78.72	46.15	21.28	Z = 2.2721 *p* = 0.0231
D	0.665 (0.538–0.793)	1.01	84.61	44.68	15.39	55.32	Z = 2.801 *p* = 0.0051
SUVmax	0.674 (0.54–0.808)	4.83	61.54	74.47	38.46	25.53	Z = 2.0186 *p* = 0.0435
MTV	0.781 (0.674–0.887)	5.29	80.77	68.08	19.23	31.92	Z = 1.078 *p* = 0.281
TLG	0.756 (0.633–0.878)	12.36	76.92	65.96	23.08	34.04	Z= 1.1879 *p* = 0.2349
MTV + D	0.841 (0.753–0.93)	**/**	92.31	63.83	7.69	36.17	**/**
Methods							
DKI	0.697 (0.568–0.826)	0.5	61.54	76.59	38.46	23.41	Z = −2.8808 *p* = 0.004
IVIM	0.736 (0.620- 0.853)	0.34	50	89.36	50	10.64	Z = −2.699 *p* = 0.0070
PET	0.745 (0.622- 0.868)	0.42	73.08	72.34	26.92	27.66	Z = −2.116 *p* = 0.0343
DKI + IVIM + PET	0.876 (0.796–0.955)	**/**	82.31	86.12	17.69	13.88	**/**

*MK* mean kurtosis, *MD* mean diffusivity, *SUVmax* maximum standardized uptake value, *MTV* metabolic tumor volume, *TLG* total lesion glycolysis, *ADC* apparent diffusion coefficient, *D* true diffusion coefficient, *FPR* false-positive rate, *FNR* false-negative rate

## Discussion

Regional lymph node recurrence and distant metastasis (DM) [31] are the main causes of therapy failure in NSCLC. Precisely predicting lymphovascular invasion (LVI) status is critical—as an independent poor prognostic factor, LVI shows significant correlation with disease-free survival and recurrence rates [32, 33], guiding personalized treatment strategies [34]. This study innovatively developed a predictive model using PET-IVIM-DKI multimodal imaging.

   Consistent with the prior study’s findings [19], this study found that SUVmax, MTV, and TLG were considerably greater in patients with LVI-positive NSCLC than in patients with LVI-negative NSCLC. This is because glucose metabolism is intimately linked to the tissue uptake of ^18^F-FDG. Hexokinase and glucose transporter-1 are activated during tumor proliferation to increase glucose uptake. Hypoxia (HIF-1), angiogenesis (VEGF and CD34), and the PI3K/Akt/mTOR signaling pathway may also be associated with the changes mentioned above. This finding further supports the usefulness of metabolic markers in forecasting LVI in NSCLC. Prior research on superficial [35] esophageal and breast [36] malignancies has produced similar findings. Shih et al discovered [37] that patients with advanced cervical cancer who had lymphovascular space involvement had significantly higher SUVmax values. In another study of lung adenocarcinoma, SUVmax was demonstrated to increase the net benefit [38] of PET-RS. However, multifactorial logistic regression revealed that MTV was the only independent predictor when it came to PET parameters. This could be because SUVmax only represents one voxel of the tumor’s metabolic process, but MTV is a three-dimensional measurement that represents measurements of tumor volume and metabolic activity, and can reflect the overall change in the tumor. This is consistent with earlier studies. Li et al [39], evaluating the LVI value of metabolically related parameters in predicting NSCLC, found that MTV was superior to SUVmax in terms of diagnostic performance and was the only significant independent predictor, with an optimal cutoff value of 6.4. The optimal cutoff value of MTV in this study is 5.29, which is different from the above results, and the reason for this difference may be that the subjects selected by different studies are different. In a prospective PET/MR study for stage prediction of endometrial cancer, Ironi et al [40] also showed that MTV, but not SUVmax, can be used to assess the prognostic efficacy of LVSI. In any event, it has been demonstrated that metabolism-related metrics can reliably predict LVI status in NSCLC.

ADCstand and D values are diffusion-related parameters of the mono-exponential and bi-exponential models of IVIM, respectively, both reflecting the diffusion of water molecules. Several studies have been conducted on the prediction of LVI in gastric cancer [41], cervical cancer [42], and other associated cancers. These studies’ findings indicate that ADC values and D values tended to be smaller in vasculatic invasive states. This can be explained by the fact that lymphovascular invasion is the first step in the invasion of tumor cells into lymphatic vessels or blood vessels, which raises the histiocyte density. Nonetheless, the role of ADC in predicting tumor LVI in NSCLC remains poorly investigated. To the best of our knowledge, only one study [43] has demonstrated the predictive value of ADC values in LVI and pleural invasion of NSCLC based on ADC histograms. With notable variations in ADCstand and D values across the LVI-positive and LVI-negative NSCLC groups, the results further support the argument and demonstrate the predictive value of ADC values for NSCLC LVI. However, in multivariate logistic regression analysis, unlike ADCstand, D is an independent predictor, which may be because of its imaging principle. D is the relevant parameter calculated by IVIM using a bi-exponential model for linear fitting of DWI signals with multiple b-values and independent of microcirculation, and is used to represent the diffusion of pure water molecules in voxels. Parameter D provides a more accurate reflection of the diffusion of water molecules. While previous studies have shown that perfusion parameters can also predict LVI, this study has shown the opposite results with two other studies [41, 44]. In this study, there was no significant difference between the LVI-positive and LVI-negative groups in terms of perfusion-related parameters D* and f. This may be due to the low repeatability of D* and f-values in lung diffusion imaging, as well as the lowest stability and reproducibility of D* when low b-values (< 200 s/mm^2^) are selected.

MK and MD, as the two key parameters of DKI, indicate the degree to which the diffusion of water molecules in the tissue deviates from a Gaussian distribution and the isotropic diffusion of water molecules within the tissue, respectively. Both are related to the cellular structure and the complexity [45, 46] of the microstructure of the tissue. The MK value increases with the complexity of the structure and the degree of divergence from the Gaussian distribution of the diffusion motion of water molecules. Due to variations in cell density, nuclear heterogeneity, and other factors, prior research has demonstrated that MK and MD values may accurately evaluate the histological type, grade, staging, and LVSI status of patients with cervical [47] cancer. The results of this study are consistent with the above theory. Compared to the LVI (−) group, the LVI (+) group in this study had significantly higher MK values and significantly lower MD values. This is because the diffusion of water molecules in LVI-positive tissues may be hindered, leading to increased cell structure, a higher nuclear-to-cytoplasmic ratio, and reduced extracellular space. Previous studies have also supported the role of MK and MD in NSCLC. Wang et al [48] discovered that by identifying the non-Gaussian diffusion features linked to modifications in the tumor microenvironment or tumor tissue complexity, DKI histogram analysis could not only differentiate between various pathological grades but also reflect the level of Ki67 expression. The AUCs of PET, IVIM, and DKI parameters did not differ statistically significantly in their ability to predict LVI status, and the diagnostic efficiency of tumor metabolism and diffusion measures was equivalent. Through multivariate logistic regression analysis, it was shown that the group of MTV and D yielded the highest area under the curve, more than that of any single parameter. At the same time, the combination of the three imaging methods also outperformed any single imaging method, demonstrating the synergistic capability of the two imaging modes. Therefore, when conditions permit, hybrid imaging techniques may be most beneficial in determining the patient’s condition.

This study has some limitations. First off, the sample size of our study was limited, which may result in less accurate results and could affect the robustness of conclusions. In the future, we will increase the sample size of NSCLC for in-depth research. Second, the non-small cell lung cancer samples in this study are mainly composed of adenocarcinoma tissue types. This may be because the incidence of lung adenocarcinoma is higher than that of other lung cancer subtypes, and it cannot accurately reflect the characteristics of the general population. This will lead to insufficient effectiveness of subgroup analysis and limit the generalizability and applicability of the study results. In addition, the number of LVI-positive group and LVI-negative groups is uneven, which may lead to potentially biased results. In the next step, we will increase the sample size of squamous cell carcinoma and try to balance the number of LVI-positive group and LVI-negative groups for further research. Third, despite the use of respiratory navigation strategies in this investigation, cardiac and macrovascular pulsation artifacts nevertheless influenced the display of lesions, particularly the IVIM parameters. Fourth, the evaluation of small lesions using the DKI system is challenging due to its low signal-to-noise ratio and spatial resolution. Fifth, this study is a single-center and single-model study, which leads to limited technical reproducibility and case selection bias. In the future, external validation is still needed using devices with different magnetic field strengths and in diverse clinical environments. Sixth, there has been no assessment of the risk of false positives/negative predictive value with this technique, which could raise the possibility of over-treatment. Finally, DWI based on Echo Planar Imaging (EPI) produces strong phase-direction artifacts in areas with susceptibility changes, such as the lung fields, resulting in significant image distortion.

## Conclusion

The present study demonstrates that both IVIM, DKI, and PET can be utilized to evaluate LVI status in NSCLC, with the combined diagnostic approach of MTV and D showing the highest diagnostic performance, which may provide a novel reference for clinical management.

## Data Availability

Due to privacy restrictions, raw data cannot be made available free of charge, but datasets used and/or analyzed during the current study may be obtained from the corresponding authors upon reasonable request.

## Abbreviations

ADCstand: Stand diffusion coefficient
D: True diffusion coefficient
DKI: Diffusion kurtosis imaging
DWI: Diffusion-weighted imaging
IVIM: Intravoxel incoherent motion
LVI: Lymphovascular invasion
MD: Mean diffusivity
MK: Mean kurtosis
MTV: Metabolic tumor volume
NEX: Number of excitations
NSCLC: Non-small cell lung cancer
T1WI: T1-weighted imaging
T2WI: T2-weighted imaging
TLG: Total lesion glycolysis
TR/TE: Repetition time/echo time
